# Depression and quality of life in patients with type 2 diabetes mellitus

**DOI:** 10.22088/cjim.13.2.3

**Published:** 2022

**Authors:** Mohammad Ali Bayani, Nava Shakiba, Ali Bijani, Sussan Moudi

**Affiliations:** 1 Social Determinants of Health Research Center, Health Research Institute, Babol University of Medical Sciences, Babol, Iran; 2 School of Medicine, Babol University of Medical Sciences, Babol, Iran

**Keywords:** Diabetes mellitus, Depression, Quality of life

## Abstract

**Background:**

Depression as a frequent comorbidity in patients with diabetes requires serious attention, as failure to early detect and treat it can adversely affect the patients' complications**. **This study was performed to assess the prevalence of depression and quality of life in these patients.

**Methods:**

This cross-sectional study was carried-out on adult patients with type 2 diabetes mellitus referred to the endocrinology hospital clinics affiliated to Babol University of Medical Sciences, Babol, Iran, during 2018-2019. The presence and severity of depressive symptoms in patients have been assessed by Beck Depression Inventory-II; and the quality of life was measured by SF-36 questionnaire.

**Results:**

Totally, 400 patients with type 2 diabetes mellitus (300 females and 100 males) with mean age of 55.36±11.56 years were recruited. One hundred and thirty (32.5%) had depressive symptoms. Of the patients with depression, 115 (28.8%) had mild and 15 (3.8%) had moderate depression. After entering different variables in logistic regression analysis, gender (P=0.036) and ophthalmic complications (P=0.011) showed a significant association with depressive symptoms. The patients with depression had a worse quality of life compared to those who had not depressive symptoms. Quality of life score in patients with depression was significantly lower than patients without depression (p<0.0001).

**Conclusion:**

Nearly one third of the patients with type II diabetes mellitus might have depression. Diabetic patients with depression have a worse quality of life compared to those who have not depression symptoms.

Type 2 diabetes mellitus (DM) is considered as a major contributor to the burden of diseases worldwide. Its attributable disability-adjusted life-years (DALYs) was calculated as 4750000 years in 2017 (1).

 In 2017, approximately 462 million persons (6.3% of the world's population) have been affected by diabetes mellitus; and a global prevalence rate of 6059 persons per 100,000 has been estimated. Annually, more than one million deaths can be attributed to DM alone, making it the 9^th^ leading cause of mortality (2). Depression as a common comorbidity in these patients requires serious attention, as failure to early detect and treat it can adversely affect the patients' self-care practices, treatment adherence, quality of life and other diabetes complications (3-6). Patients with DM have a 2–4 fold greater risk of comorbid depression when compared to individuals without diabetes (7, 8). A systematic review and meta-analysis revealed that nearly 28% of patients with DM might have depression and this value is higher in Asia (32%) compared to Europe (24%), Africa (27%), America (28%) and Australia (29%) (3).

The overall prevalence of depression in Iranian people with diabetes was represented as 54% (95% CI: 47.32–60.70); furthermore, the prevalence of depression in women (56.3%) was reported higher than that of men (41.1%) (5). Quality of life (QOL) refers to the perception of physical, emotional, and social status that everyone has about himself. A primary goal in early diagnosis and treatment of patients with diabetes is QOL; and quality of life in these patients becomes worse when any comorbidity coexists or complications start to develop (9, 10). 

 Although the precise factors that deteriorate the QOL in patients with diabetes have not been determined, some previous studies indicated that the patient’s age, type of DM, ethnicity, economic status, educational level, and social support; medications (especially injections) administered by the patients, diabetes-related complications and psychological factors may interfere in the QOL for these individuals (11, 12). 

Either depression or diabetes are correlated with morbidity and mortality, and when these two diseases are concomitant in a patient, the risk for poor blood glucose control, developing complications, and related cost increase (4). 

Previous studies that examined the prevalence of depression in patients diagnosed with diabetes reported different results. A research showed that the prevalence of depression among Iranian patients with diabetes ranged from 13.7% to 87% (5). Considering the fact that the initial step in planning to control depression in diabetic patients is the accurate identification of the current situation (13), we conducted this study to assess the prevalence of depression and quality of life in these patients.

## Methods

This descriptive-analytic research was conducted as a cross-sectional study during 2018-2019 on adult patients with type 2 diabetes mellitus that referred to the endocrinology hospital clinics affiliated to Babol University of Medical Sciences, Babol, Iran. Diabetes mellitus was diagnosed by the study endocrinologist, and if the patient had two fasting blood glucose ≥126 mg/dL or other diagnostic criteria related to DM (2). 

These patients were selected by convenience sampling. They were evaluated for depression and quality of life if they desired to participate in the study. Any cognitive impairment, dementia, and mental retardation have been considered as exclusion criteria. Based on the results of previous studies in Iran, consider 50% prevalence of depression in patients with diabetes (14); with 95% confidence level and d = 0.05, sample size was calculated as 400 individuals. Beck Depression Inventory-II (BDI) and SF-36 questionnaire were used for data collection. In addition sociodemographic and clinical characteristics, (age, gender, occupational and marital status, how to live with family members, education level, approximate monthly income and treatment cost, duration of diabetes, type of antidiabetic drugs, and previously diagnosed complications of diabetes) were collected through an interview. After initial assessment with BDI, suspected patients with depressive symptoms (BDI score ≥16) were referred to the psychiatrist for further examination. 

Beck Depression Inventory-II is a 21-item self-reporting questionnaire that examines the presence and severity of depressive symptoms in patients. Each question has four options for selection; and the subject should select one option which is most compatible with his or her current condition, and accordingly, the rating of each question is 0-3. Total score indicates the status and severity of depression in the patient: 1–15 means without depression, 16–30 with mild depression, 31–46 moderate; and 47–63 means severe depression symptoms. The validity and reliability of this questionnaire has been approved in adult populations (15, 16). 

SF-36 is a thirty-six item questionnaire that measures the quality of life in eight scales including physical functioning; emotional well-being; physical pain; general health; energy and fatigue; social functioning; physical health problems; and emotional health problems. Each scale is directly transformed into a score from 0 to 100. The lower the score demonstrates the more disability. The validity and reliability of this questionnaire has been evaluated in Iranian adult population (17). Diabetes-related complications including ophthalmic, cardiovascular, renal and other complications were recorded by the research endocrinologist, based on the patient's medical examinations, laboratory or imaging findings and/or prescribed medications. 

Chi-square, t-test and logistic regression analysis were used for data analysis in SPSS-18 software package. This study has been approved by the Ethics Committee of Babol University of Medical Sciences with registration code MUBABOL.REC.1394.299. 

## Results

In the present study, 400 patients with type 2 diabetes mellitus were recruited. The mean age of participants was 55.36±11.56 years and most of them (64.3%) were under 60 years old. Their sociodemographic and clinical characteristics are presented in table 1. The results showed that 300 (75.0%) of patients were females; 363 (90.8%) married; 80.5% a undergraduate, 204(51.0%) individuals were living in rural regions. One hundred and two (25.5%) patients had diabetes for about 15 to 20 years. In addition, 48 (12.0%) subjects had a history of psychiatric diseases; and 41 (10.2%) patients reported a family history of psychiatric disorders. Of the 400 patients with DM, 130 (32.5%) had depressive symptoms. Of the patients with depression, 115 (28.8%) had mild and 15 (3.8%) with moderate depression. None of the individuals had severe depression. Table 2 represents the distribution of sociodemographic variables among DM patients with and without depression. This table shows that 86.9% of women and 13.1% of men with type 2 diabetes had depression (P=0.0001).

 A significant association was observed between occupational status (0.030), marital status (0.046), and treatment cost (0.004) with depression. Seventy-nine (60.8%) patients with depression had monthly income ranging from 100 to 200 dollars; however, in the non-depressed group 167 (61.9%) were in this range of monthly income (p>0.05). 

The association of depression symptoms with diabetes-related health complications has been presented in table 3. A significant association has been found between ophthalmic (P=0.007), and cardiovascular (P=0.001) complications with depression. Furthermore, the patients with a previous history of hospital admission related to DM had a higher prevalence of depression (P=0.001). However, after entering the variables of gender, marital and occupational status, the approximate monthly cost of treatment, ophthalmic, cardiovascular and other complications of diabetes, and previous history of hospital admission related to DM in logistic regression analysis, the results showed a significant association between depression symptoms and gender (P=0.036), and ophthalmic complications (P=0.011). Other characteristics including marital status (P=0.651), occupational status (P=0.210), cardiovascular complications (P=0.282), previous history of hospital admission (P=0.132), and treatment costs (P=0.457) had no significant association with depression symptoms. The mean score of the eight categories of quality of life was 61.96±30.59 for physical functioning; 55.54±19.24 emotional well-being; 57.66±24.49 physical pain; 47.56±15.59 general health; 52.91±18.20 energy and fatigue; 59.57±24.34 social functioning; 53.93±40.99 physical health problems; and 49.75±42.14 for emotional health problems. The highest and lowest scores were related to physical functioning (61.96) and general health (47.56), respectively. The association between depression and quality of life in patients with DM is presented in table 4. The results showed that quality of life score in patients with depression was significantly lower than patients without depression (p<0.0001). 

**Table 1:** Baseline sociodemographic and clinical characteristics of patients diagnosed with type 2 diabetes mellitus (N=400)

**Variable**	**Frequency**	
	**Number**	**Percent**
**Gender** Male Female	100 300	25.0 75.0
**Marital status** Married Single	363 37	90.8 9.2
**How to live with family members** Living with family members Living alone	379 21	94.8 5.2
**Occupation** Housekeeper Employee Others	289 28 72	72.3 9.5 18.2
**Monthly income (in dollars) ** < 50 50-100 100-200 >200	37 84 246 33	9.3 21.0 61.5 8.2
**Type of antidiabetic drugs the patient was taking** Oral Injecting	291 109	72.8 27.2
**Approximate cost of treatment in one month (in dollars)** <10 10-25 >25	161 171 68	40.3 42.7 17.0
**Diabetes duration (year)** <1 1-5 5-10 10-15 15-20 >20	2 32 94 107 102 63	0.5 8.0 23.5 26.7 25.5 15.8
**Complications of diabetes which have been diagnosed** Ophthalmic Cardiovascular Renal Others	153 140 88 72	38.3 35.0 22.0 18.0

**Table 2:** Association of depressive symptoms with sociodemographic and clinical characteristics of patients with diabetes mellitus

**Variable**	**Depressive Symptoms**		**Crude ** **P-value**
	**Yes** **N(%)** **N=130**	**No ** **N(%)** **N=270**	
**Gender** Male Female	17 (13.1) 113 (86.9)	83 (30.7) 187 (69.3)	0.0001
**Living region** Urban Rural	71 (54.6) 59 (45.4)	125 (46.3) 145 (53.7)	0.119
**Marital status** Married Single	114 (87.7) 16 (12.3)	249 (92.2) 21 (7.8)	0.046
**How to live with family members** Living with family members Living alone	119 (91.5) 11 (8.5)	260 (96.3) 10 (3.7)	0.460
**Occupational status** Housekeeper Employee Others	105 (80.8) 8 (6.1) 17 (13.1)	184 (68.2) 30 (11.1) 56 (20.7)	0.030
**Type of antidiabetic drugs the patient was taking** Oral Injecting	89 (68.5) 41 (31.5)	202 (74.8) 68 (25.2)	0.181
**Approximate cost of treatment in one month (in dollars)** <10 10-25 >25	37 (28.5) 67 (51.5) 26 (20.0)	124 (45.9) 104 (38.5) 42 (15.6)	0.004
**Previous history of psychiatric illness in the patient ** Yes No	15 (11.5) 115 (88.5)	33 (12.2) 237 (87.8)	0.840

**Table 3:** Association of depressive symptoms with diabetes related health complications

**Diabetes related health complications**	**With depressive symptoms** **N(%)** **N=130**	**Without depressive symptoms** **N(%)** **N=270**	**Crude** **p-value**
**Ophthalmic complications** Yes No	62 (47.7) 68 (52.3)	91 (33.7) 179 (66.3)	0.007
**Cardiovascular complications** Yes No	60 (46.2) 70 (53.8)	80 (29.6) 190 (70.4)	0.001
**Renal complications** Yes No	26 (20.0) 104 (80.0)	62 (23.0) 208 (77.0)	0.503
**Other complications** Yes No	32 (24.6) 98 (75.4)	40 (14.8) 230 (85.2)	0.017
**Previous history of hospital admission because of diabetes and its complications** Yes No	45 (34.6) 85 (65.4)	51 (18.9) 219 (81.1)	0.001

**Table 4:** The association between quality of life score and depression in patients with type-2 diabetes mellitus

**Quality of life scales**	**With depressive symptoms (N=130)** **Score (Mean±SD)**	**Without depressive symptoms (N=270)** **Score (Mean±SD)**	**p-value**
Physical functioning	51.23±28.82	67.12±30.12	<0.0001
Emotional well-being	41.52±13.35	62.30±17.97	<0.0001
Physical pain	46.25±21.21	63.16±24.09	<0.0001
General health	38.61±14.31	51.87±14.32	<0.0001
Energy and fatigue	42.23±15.89	58.05±16.99	<0.0001
Social functioning	48.97±22.72	64.67±23.47	<0.0001
Physical health problems	40.76±40.48	60.27±39.78	<0.0001
Emotional health problems	31.02±36.70	58.76±41.69	<0.0001

## Discussion

In our research, 32.5% of 400 patients with DM had mild to moderate depression symptoms and no patient had severe depression. Depression was more prevalent in females compared to males. Khaledi et al. reported a global estimated prevalence of comorbid depression of 28% (23% of males and 34% of females) in patients with type 2 diabetes, through a systematic review and meta-analysis (3). Sartorius´ study indicated a prevalence of 10-15% of depressive disorders in diabetic patients, which was nearly two times higher than the prevalence of depression in nondiabetic individuals (13). Kreider reported that the presence of major depressive disorder in people with DM may be up to three times more common than in the general population (18). Nanayakkara et al.’s study in Australia revealed that 29% of adult patients with type 2 DM had likely depression, 7% had high diabetes distress, and 5% had both of them (8). This difference in results might be due to the study population, sociodemographic and clinical characteristics, definitions and tools that have been used to evaluate the depressive disorders in DM patients. Gender had a significant association with depressive symptoms in DM patients, however, marital status, occupation and treatment costs did not show a significant association. Women are at higher risk than men for developing depression, with or without diabetes (13, 19). A review article indicated that the risk of depression in women with diabetes can be twice that of nondiabetic peers (13). This could be related to this fact that women experience more negative life events, and experience significant hormonal changes during pregnancy, postpartum and menopausal period (13). In addition to, women appear to be more sensitive to socioeconomic predictors, such as education, occupation, and income for the future development of diabetes and its related depression (20). 

Social factors such as low educational level, occupation, inadequate income, unhealthy lifestyle behaviors and social disparities can be related to a higher risk of depression in patients with diabetes (20). Also, diabetes and its complications have a significant economic impact on people with diabetes and their families through direct medical costs and loss of work and wages. The World Health Organization(WHO) represented the hospital and outpatient care as the major cost drivers in diabetic patients; furthermore, another contributing factor which should be notified is the difficulty to access and the rise in cost for insulins, especially in low and middle income countries (19). Sartorius reported that treatment cost of diabetes when depression coincides can be 4.5 times higher than the treatment of diabetes alone (13). This difference in results can be attributed to different study population, demographic and socioeconomic characteristics of the recruited patients. In this research, depressive disorder was more prevalent in patients who had diabetes-related cardiovascular complications. Prospective studies demonstrated that depressive disorders was associated with increased risk of macrovascular and microvascular consequences of diabetes, even after adjusting for DM severity and self-care behaviors (18). Rees et al. reported moderate or severe visual impairment, and severe asymptomatic nonproliferative or symptomatic proliferative diabetic retinopathy as independent risk factors for depressive symptoms in persons with diabetes (21). Disability and difficulties in caring for and treatment of cardiovascular and ophthalmic outcomes of diabetes can justify a higher prevalence of depression in DM patients with these consequences. 

This study indicated that the patients with depression had a worse quality of life compared to those who had not depressive symptoms. All of the SF-36 subscales (including physical functioning, emotional well-being, physical pain, general health, energy and fatigue, social functioning, physical health problems and emotional health problems) showed a lower score in DM patients with depression. Similar to our result, Derakhshanpour et al. examined 330 diabetic patients via Beck Depression Inventory and the quality of life questionnaire recommended by the World Health Organization (WHOQOL-BREF), and found patients with depression had a significant lower score than non-depressed diabetic patients (14). Altinok et al. assessed the quality of life in 440 adult patients with DM by SF-36 questionnaire and reported that the mean scores of all SF-36 subscales were significantly lower in females than those of male individuals. The patients with longer duration of diabetes, or using injecting antidiabetics had lower scores of quality of life. Also, physical function score of the patients with no diabetes-related health complications was significantly higher than those with two and more complications (22). 

Trikkalinou et al. demonstrated that diabetes-related complications, especially coronary artery disease, renal failure, blindness, and sexual dysfunction deteriorate the quality of life in diabetic patients (9). Depression can disturb emotions, cognition, and behaviors of diabetic patients. When depression is diagnosed in a diabetic patient, both diseases should be treated at the same time, as the response to medication is usually seen within 2-4 weeks for antidepressants, and the improvement in glycemic control and serum level of HbA1C needs several months. Furthermore,a better mood might result to a much better patient’ s adherence to diabetic treatment protocol (18). 

In this study, no structured clinical interview was performed and the used questionnaires were self-reported. It can be represented as the most important limitation of this research. Longitudinal studies are suggested to examine the actual impact of diabetes mellitus on quality of life and the different aspects of patients´ mental health. 

In conclusion nearly one third of the patients with type II diabetes mellitus may have depression. More depression symptoms are expected to occur in patients with ophthalmic or cardiovascular complications. Diabetic patients with depression have a worse quality of life compared to those who have not depressive disorder. 
